# Bilateral multiple serous retinal detachments after treatment with nivolumab: a case report

**DOI:** 10.1186/s12886-020-01495-w

**Published:** 2020-06-08

**Authors:** Reina Miyamoto, Hiroyuki Nakashizuka, Koji Tanaka, Yu Wakatsuki, Hajime Onoe, Ryusaburo Mori, Akiyuki Kawamura

**Affiliations:** Division of Ophthalmology, Department of Visual Sciences, Nihon University School of Medicine, Nihon University Hospital, 1-6 Kandasurugadai, Chiyoda-ku, Tokyo 101-8309 Japan

**Keywords:** Immune checkpoint inhibitors, Nivolumab, Fundus autofluorescence, Serous retinal detachment

## Abstract

**Background:**

Immune checkpoint inhibitors have recently been widely used for advanced cancers and are known to cause ocular complications. We herein report a case developing bilateral serous retinal detachments, without ocular inflammation, after starting nivolumab treatment.

**Case presentation:**

A 73-year-old man was referred to our hospital, having become aware of metamorphopsia 2 months after starting nivolumab (anti-programmed cell death protein 1 monoclonal antibody) for malignant melanoma of the nasal cavity. The initial corrected visual acuity of the right eye was 20/20, and that of the left eye was 20/16. There were no inflammatory findings in the anterior segment or the vitreous. Vitelliform lesions were found in the macular area of both ocular fundi, consistent with serous retinal detachment and subretinal deposits. Swept source optical coherence tomography showed diffuse thickening of the outer photoreceptor segment and thickening of the choroid. Two months after the initial diagnosis, multiple vitelliform lesions were noted, and the fundus findings had worsened. Indocyanine green fluorescein angiography showed delayed inflow in the peripapillary and posterior pole regions in the early phase of imaging. Fundus autofluorescence showed hyperautofluorescence consistent with most of the vitelliform lesions on color fundus photography.

**Conclusions:**

Nivolumab may have impaired the pumping and phagocytosis functions of retinal pigment epithelial cells, resulting in bilateral serous retinal detachments and thickening of the photoreceptor outer segment. This is the first case report, to our knowledge, describing multiple bilateral serous retinal detachments and outer segment thickening without inflammation in a patient treated with nivolumab.

## Background

Recently, immune checkpoint inhibitors have been widely used for advanced cancers. Among these agents, nivolumab is one of the earliest to be developed and is used to treat various cancers, including renal cell carcinoma, malignant melanoma, and Hodgkin lymphoma [1].

Immune checkpoint inhibitors modulate immune control mechanisms activating immunity and thereby indirectly attacking cancer cells. Cancer cells express PD-L1 (programmed death protein ligand 1), which is a ligand for PD-1 (programmed death protein1) expressed on activated T cells. Upon binding of PD-1 and PD-L1, activated T cells are inactivated, and cancer cells proliferate. Nivolumab preparations are antibodies to PD-1 and are believed to prevent the growth of cancer cells by stimulating T-cell activation. The different types and subclasses of immune checkpoint inhibitors are each associated with several characteristic immunity-related complications [1]. Among ocular complications, dry eye (< 1–5%), uveitis-like symptoms (< 1%), and Vogt-Koyanagi-Harada (VKH) disease (incidence unknown) have been reported[2]. The possibility of developing VKH disease is indicated by nivolumab targeting the same antigens as the those of the melanocytes comprising malignant melanoma and melanocytes of the choroid [3–6].

We herein report a patient with bilateral serous retinal detachments and photoreceptor outer segment thickening, without evidence of uveitis such as in VKH disease, thought to have been caused by nivolumab treatment. Our search of the literature yielded no similar cases.

## Case presentation

A 73-year-old Japanese man was referred to our hospital with a chief complaint of metamorphopsia affecting both eyes.

**Fig. 1:**
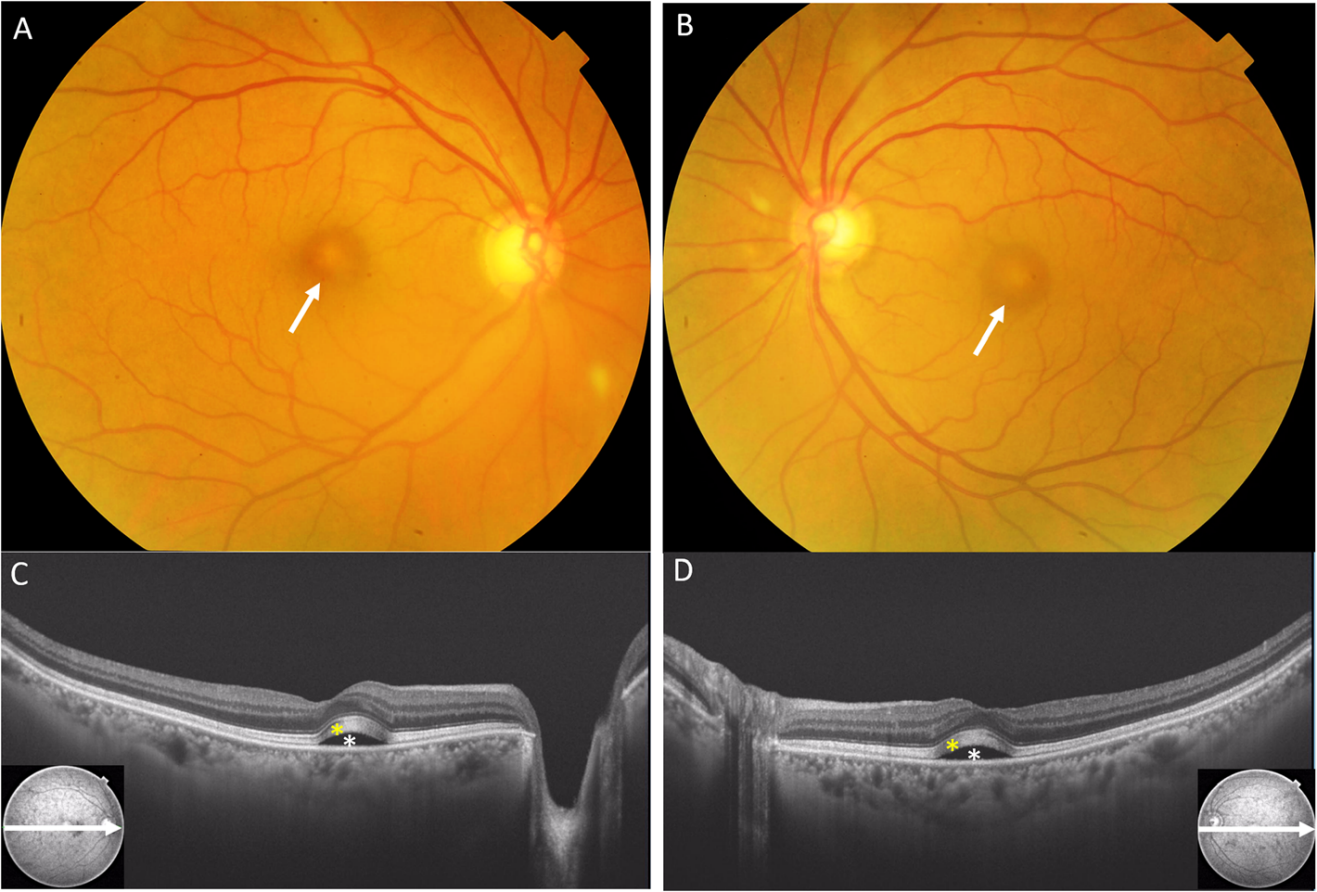
The findings at initial presentation, BCVA in the right eye 20/20, left eye 20/16. Fundoscopy revealed vitelliform lesions in the macular area of both eyes (**a**, **b**: white arrow), and OCT showed bilateral serous retinal detachments (**c**, **d**: white asterisk). Diffuse lamellar thickening in the photoreceptor outer layer (**c**, **d**: yellow asterisk) and choroidal thickening were detected by SS-OCT

In 2014, the patient had been diagnosed with malignant nasal melanoma stage 4 including metastases to the lung, esophagus, and bone, and nivolumab at a dose of 3 mg/kg every 2 weeks was started in February 2017. Two months after starting this regimen, he became aware of metamorphopsia in both eyes. The findings at initial presentation were best corrected visual acuity (BCVA) in the right eye 20/20, left eye 20/16. Intraocular pressure was 10 mmHg in both eyes. There were no inflammatory cells in the anterior segment or the vitreous. Fundoscopy revealed vitelliform lesions in the macular area of both eyes, and swept source optical coherence tomography (SS-OCT, Topcon DRI OCT-1 Atlantis) showed bilateral serous retinal detachments. Diffuse lamellar thickening in the photoreceptor outer segment and choroidal thickening were also observed (Fig. 1).

**Fig. 2:**
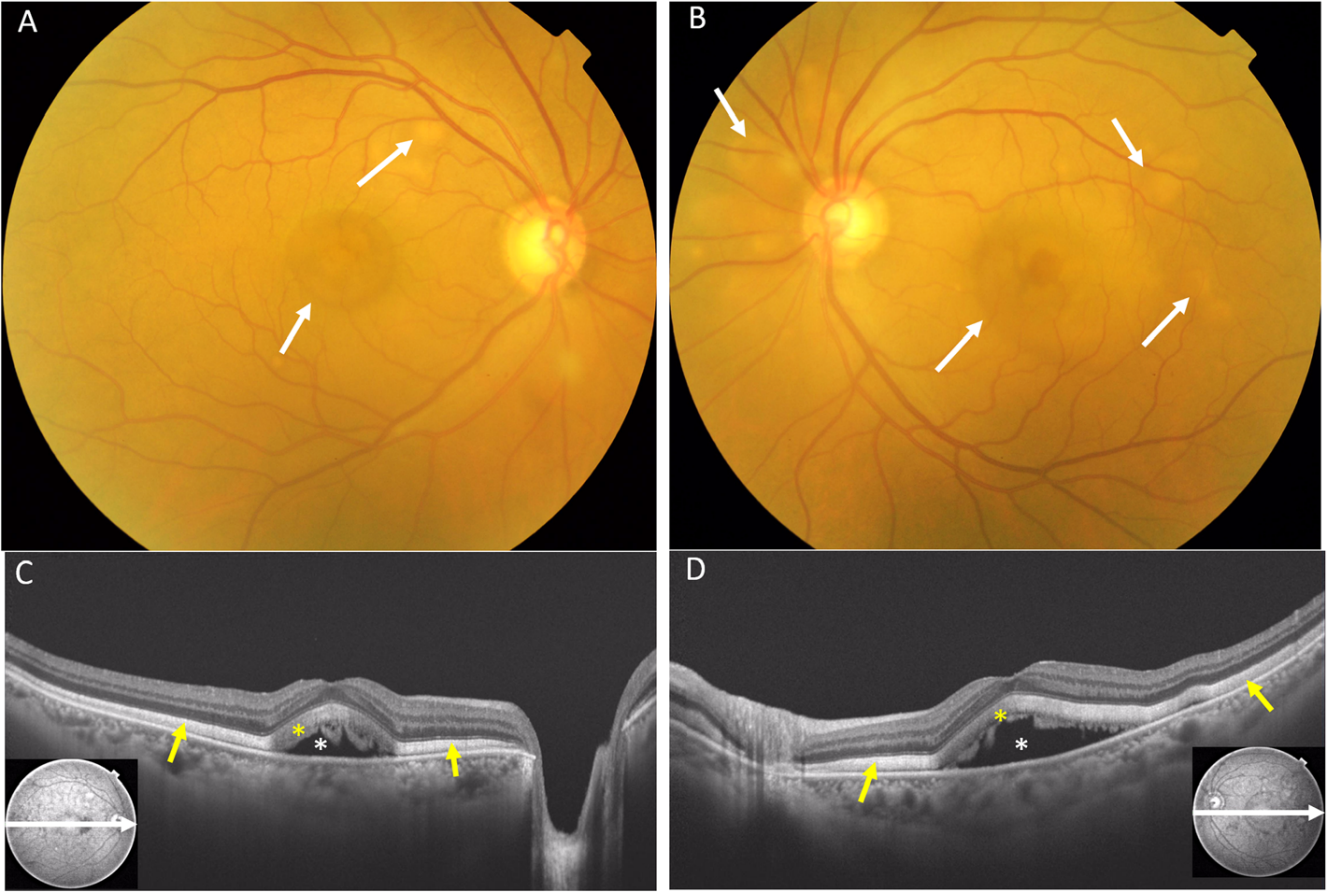
The findings at 2 months after the initial presentation, BCVA in the right eye 20/20, left eye 20/20. The vitelliform lesions in the fundus became more numerous and tended to enlarge (**a**, **b**: white arrows). SS-OCT showed worsening of the serous retinal detachments (**c**, **d**: white asterisk) and diffuse lamellar thickening in the photoreceptor outer layer (**c**, **d**: yellow asterisk). The hyperreflective band became wider and more prominent even in the regions without retinal detachment (**c**, **d**: yellow arrows). The choroidal thickness reached 341 μm in the right eye and 370 μm in the left eye

**Fig. 3:**
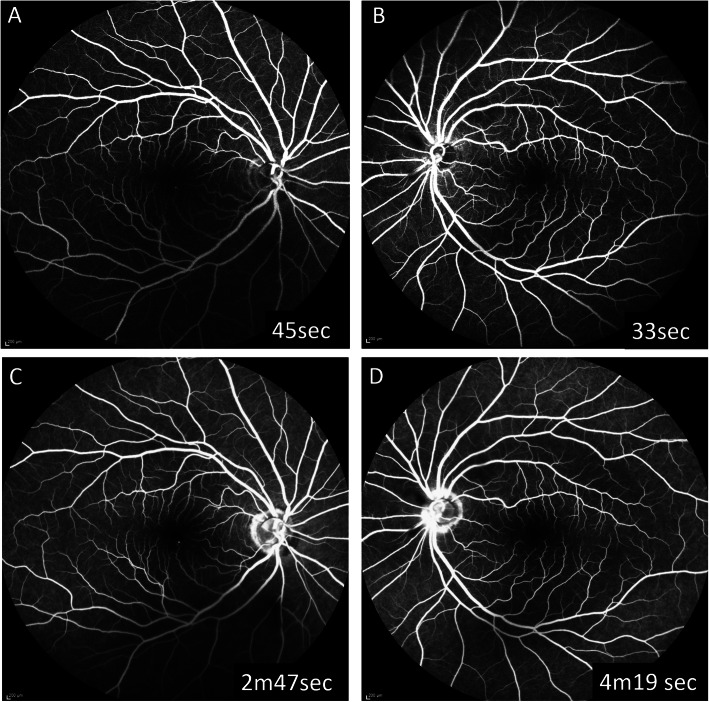
FA at 2 months after the initial presentation. On FA, no choroidal flush was observed in the early phase, but there was no delay in entry into the retinal vessels (**a**, **b**). In the late phase of FA, there was neither pooling nor obvious leakages, such as the pinpoint leakage which is characteristic of eyes with metastatic choroidal carcinoma (**c**, **d**)

**Fig. 4:**
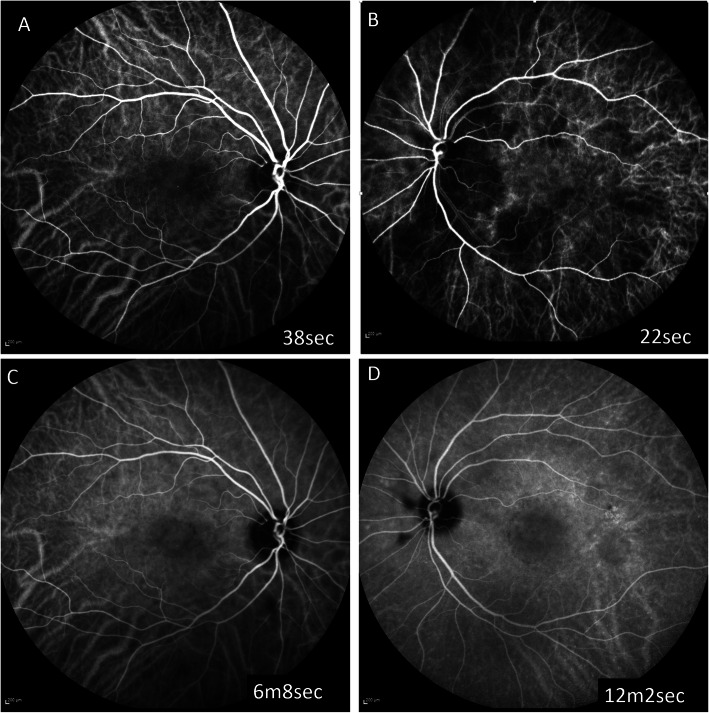
IA at two months after the initial presentation. IA showed delayed inflow centered on the optic disc and posterior pole of the fundus in the early phase of imaging (A, B), and a corresponding blockage in the serous retinal detachment region in the late phase of imaging (C, D)

**Fig. 5:**
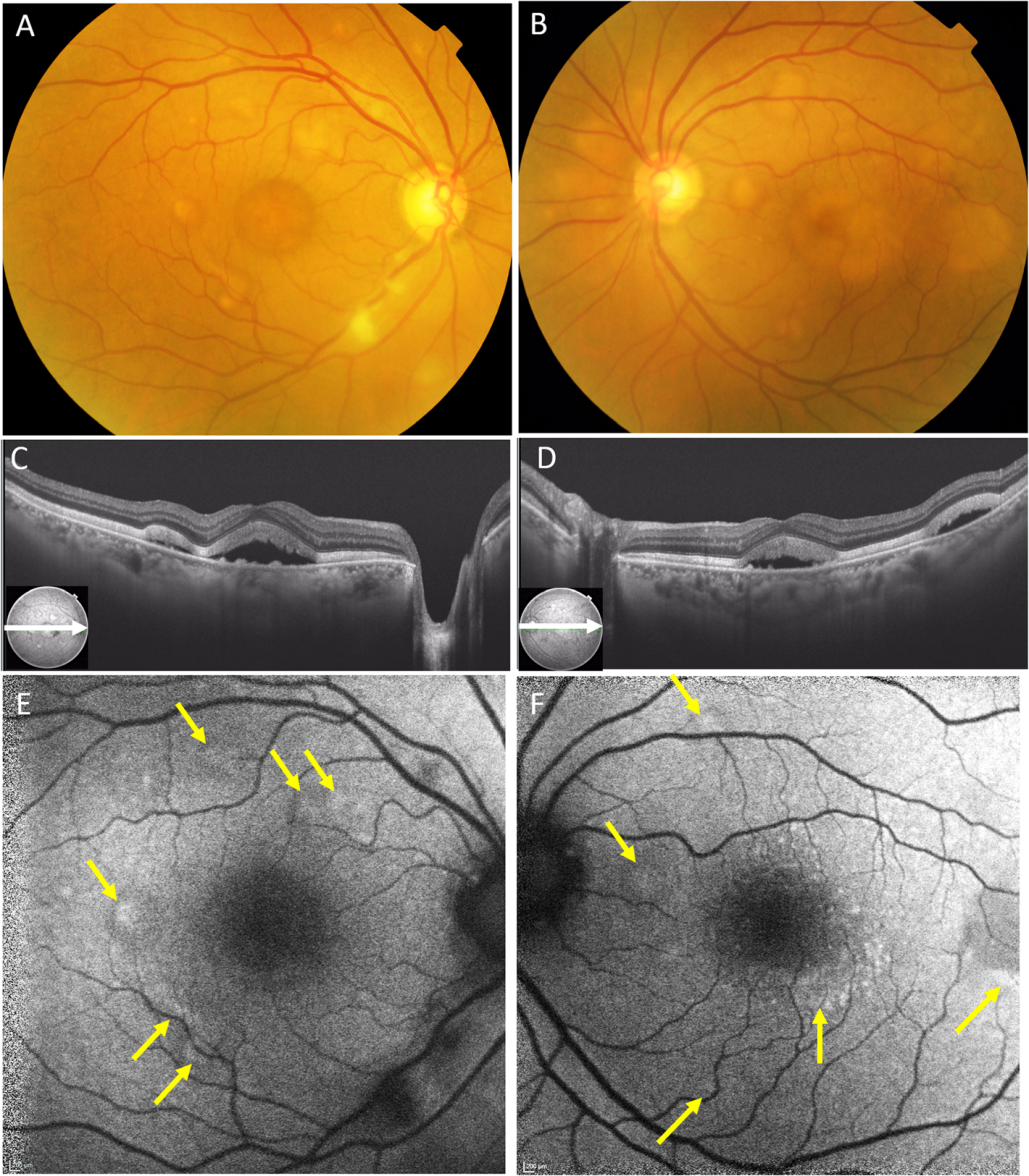
The findings at 3 months after the initial presentation, BCVA in the right eye 20/20, left eye 20/20. The vitelliform lesion had further enlarged (**a, b**). SS-OCT showed that choroidal thickness had increased to 365 μm in the right eye (**c**) and 383 μm in the left eye (**d**). FAF showed hyperautofluorescence consistent with most of the vitelliform lesions on color fundus photography (yellow arrows), the exception being the foveal area which showed hypoautofluorescence due to blockage by xanthophyll and serous retinal detachment (**e, f**)

Two months later, though the BCVA remained good in both eyes, there were more vitelliform lesions in the fundus and they showed a tendency for enlargement. Serous retinal detachment and diffuse lamellar thickening in the photoreceptor outer segment had worsened bilaterally. A broad hyperreflective band was more prominent even in the regions without retinal detachments. The choroidal thickness had also increased in both eyes (Fig. 2). On fluorescein angiography (FA, Spectralis®, Heidelberg Engineering Inc., Heidelberg, Germany), no choroidal flush was observed in the early phase, but there was no delay in entry into the retinal vessels. In the late phase of FA, there was no pooling or obvious leakages (Fig. 3). Indocyanine green fluorescein angiography (IA, Spectralis®) showed delayed inflow centered on the optic disc and posterior pole of the fundus in the early phase of imaging, and a consistent block in the serous retinal detachment region in the late phase of imaging (Fig. 4). Three months after the initial presentation, the vitelliform lesions had further increased in both size and number. The choroidal thickening was also more prominent. Fundus autofluorescence (FAF) was examined (Spectralis®). The fundus showed hyperautofluorescence consistent with most of the vitelliform lesions on color fundus photography (Fig. 5). Since the patient’s BCVA was still good, systemic treatment was prioritized, and no ophthalmologic treatments were administered. The patient was unable to continue visiting our hospital due to deterioration of his systemic condition. He died 2 months after the last visit to our facility.

## Discussion and conclusions

The patient described herein had multiple vitelliform lesions accompanied by serous retinal detachments, photoreceptor outer segment thickening and choroidal thickening, all of which manifested during nivolumab treatment for metastatic melanoma. Retinal pigment epithelial (RPE) cells reportedly express PD-L1, which interacts with PD-1[2, 7]. T cells were activated by nivolumab, possibly promoting attacks on the patient’s own RPE cells expressing PD-L1 and thereby impairing RPE functions. On FAF, hyperautofluorescence was recognized as corresponding to most of the sites of the vitelliform lesions in the fundus. This indicates that there is lipofuscin deposition in the RPE cells. We thus speculated that decreased RPE pumping function resulted in serous retinal detachment and elongation of the photoreceptor outer segment due to a decrease in the phagocytic function of RPE cells. This mechanism might account for the nivolumab-related maculopathy in our case.

However, there are several diseases that must be considered in the differential diagnosis as they can produce pathology similar to that in our case. As mentioned above, VKH disease is the one of the most important differential diagnoses to consider in our present case. Since neither anterior segment inflammation nor fluorescent leakages were observed on angiographic studies in this case, the mechanism underlying serous retinal detachment was considered to differ from that previously reported for the onset of VKH disease caused by nivolumab [3–6]. However, thickening of the choroid and delayed choroidal filling have been observed, raising the possibility of overlapping pathologies.

Another possible diagnosis is the paraneoplastic acute exudative polymorphous vitelliform maculopathy (AEPVM) seen in patients with metastatic melanoma [8, 9]. AEPVM is a rare disorder associated with multifocal areas of serous detachment and subretinal accumulation of hyperautofluorescent yellowish material in the posterior pole [10]. Although the underlying mechanism is not fully understood, an inflammatory or immune-mediated process is suspected. Koreen et al. reported that paraneoplastic autoimmune reaction against the RPE, with peroxiredoxin 3 as the putative antigen, might be a cause of AEPVM [9]. Furthermore, AEPVM triggers are associated with trauma, infectious diseases, and idiopathic processes, as well as melanoma and carcinoma. Therefore, paraneoplastic AEPVM is thought to be a variant of melanoma-associated retinopathy. There is a recent report describing paraneoplastic AEPVM as closely resembling the course of our case in their patient treated with a combination of vemurafenib (a BRAF inhibitor) and pembrolizumab, which has the same mechanism of action as nivolumab, administered as an anti-PD-1 antibody agent for metastatic melanoma [8]. Discontinuation of vemurafenib and introduction of difluprednate and dorzolamide led to a gradual resolution of AEPVM. The authors concluded that, in assessing the cause of AEPVM, it is difficult to differentiate between a direct association with the use of vemurafenib as treatment for metastatic melanoma and indirect triggering of an autoimmune-paraneoplastic process. The authors also considered the possibility that pembrolizumab might have potentiated an immune-related adverse event in their case, because checkpoint inhibitors can cause or exacerbate autoimmune diseases including melanoma-associated retinopathy. Since the melanoma in our case was treated with nivolumab alone, the causal mechanism might have been similar to that associated with pembrolizumab, another anti-PD-1 antibody, rather than the mechanism of action of vemurafenib.

There have also been reports of ipilimumab, an antibody inhibiting the cytotoxic T lymphocyte (T-cells)-associated antigen 4 (CTLA-4) molecule, i.e. another immune-checkpoint inhibitor, showing OCT findings closely resembling those of our case [11, 12]. The RPE cells express CD68 molecules which interact with CTLA-4 [2]. Therefore, ipilimumab might trigger T-cells to attack the host’s own RPE resulting in ipilimumab-related retinopathy.

We have herein reported the first case, to our knowledge, of serous retinal detachment and photoreceptor outer segment thickening, with no inflammatory findings, caused by treatment with nivolumab alone. The patient was unable to continue visiting our hospital due to deterioration of his systemic condition. Therefore, the final outcome of his ocular condition is unknown. Had ocular symptoms or fundus findings worsened, discontinuation of nivolumab and treatment with topical dexamethasone would have be considered, in consultation with the physician managing overall care.

The indications for immune checkpoint inhibitors are expected to expand as the variety of these agents increases. It should be noted that not only those previously reported, but also a wide variety of ocular complications may occur.

## Data Availability

Not applicable.

## Abbreviations

BCVA: Best corrected visual acuity
CTLA-4: Cytotoxic T lymphocyte (T-cell)-associated antigen 4
FA: Fluorescein angiography
FAF: Fundus autofluorescence
IA: Indocyanine green angiography
OCT: Optical coherence tomography angiography
PD-1: Programmed death protein1
PD-L1: Programmed death protein ligand
SS-OCT: Swept source optical coherence tomography
